# Book Review

**Published:** 1894-09

**Authors:** 


*The Human Element in Sex.* By Dr. ELIZABETH BLACKWELL. New Edition. Pp. 76. London: J. & A. CHURCHILL. 1894.

The questions involved in the relationship of the sexes are so important and intricate that few writers have felt competent to publish their views on the subject. Great tact and knowledge combined with deep thought and long experience can alone justify anyone in the attempt; without these it is far better to leave such matters alone.

The psychical as well as the physical significance of the sexual act, the injurious effect of impurity on the national life, and the duty of parents in such matters, are dealt with in this small book, and there is no harm in emphasising these things from time to time. But when we are told that menstruation and the involuntary emission of semen are strictly analogous, that there is no rudiment in the male corresponding to the uterus in the female, and that circumcision in boys must be entirely condemned as a barbarous mutilation, we feel that we cannot look upon the authoress as a beneficent light to guide us through troubled waters, however good her intentions may be.

*On Chorea.* By OCTAVIUS STURGES, M.D. Second Edition. Pp. xi., 188. London: JOHN BALE & SONS. 1893.—The purpose of this well known work is to show that chorea is in a large measure a disease of function due to preventible causes; and the author has somewhat modified his earlier views as to the rheumatic connection of chorea and the significance of its earlier phenomena. "The belief in its rheumatic connection is now firmly rooted in this country: we no longer seek to verify the connection, but are rather occupied in constructing theories to explain it." The chief therapeutic agents on which the author relies are sufferance, patience, cheerful anticipation, a firm unshaken purpose, and self-reliance: such remedies as are difficult to find, and more so to provide. In his belief there is no drug which is directly serviceable in the sense of having some specific action.

*Medicated Baths in the Treatment of Skin Diseases.* By LESLIE PHILLIPS, M.D. Pp. 103. Birmingham: CORNISH BROTHERS. 1893.—This little volume on a branch of balneo-therapeutics shows what can be done at home by the use of artificial baths in the treatment of skin diseases. No attempt is made to make the book serve as a guide to the use of natural baths. The selection and natural use of such rational baths as can be readily prepared either in hospital or in private practice is much to be commended, in place of the banishment of the patient to distant and foreign health-resorts where the natural baths are provided.

*Outlines of Gynæcological Diagnosis.* By N. T. BREWIS, M.B. Pp. 68. Edinburgh: WILLIAM F. CLAY. 1894.—This is a handy little book for "students and practitioners"; and teaches on careful lines the principles of a subject with which students frequently have some difficulty. There is an obvious error on page 11, where we are told "that the umbilicus lies over the second last lumbar vertebra;" and those who object to hybrids will find cause for complaint in the use of the word "monolocular" as opposed to "multilocular" on page 14. The work is usefully illustrated from Schultze, and fulfils its object; the remarks on examination *per rectum* are well worth attention, for this method of examination is too frequently neglected in gynæcological practice. There is no index.

*The Use of Antiseptics in Midwifery.* By ROBERT BOXALL, M.D. Pp. 35. London: H. K. LEWIS. 1894.—The two lectures which form this pamphlet are excellent value for the small sum of one shilling, as they call attention to the possibilities of antiseptics in midwifery cases. Dr. Boxall quotes statistics which show that the death rate from puerperal fever during the last forty years has not decreased in London, and has apparently increased in the provinces. Dr. Boxall thinks that these lamentable conditions are to be attributed to the fact that no approach towards the general adoption of *efficient* antiseptic measures has yet been made. He prefers perchloride of mercury; and he lays down the rule that this should never be used except by a medical man who is fully alive to its dangers. Imagine that everything you touch is coated with lampblack and act accordingly, is really the keynote to this small work; and he winds up by saying: "Not only use antiseptics, but use them with intelligence and care."

*Infectious Diseases: Notification and Prevention.* By LOUIS C. PARKES, M.D. Pp. xvi., 185. London: H. K. LEWIS. 1894.—The author has selected and re-arranged in consecutive form those portions of the Public Health and allied Acts which relate to infectious diseases. These alone should be of great service to the private practitioner in his relations with public authorities involved by the wide application of the Notification Act. In addition, chapters on infectious disease, school outbreaks, home isolation, disinfection, and kindred subjects much extend the value of the book. The size and flexible binding (which is extremely neat) render it a handy and convenient pocket companion.

*On Common Neuroses; or, The Neurotic Element in Disease, and its Rational Treatment.* By JAMES FREDERIC GOODHART, M.D. Second Edition. Pp. 135. London: H. K. LEWIS. 1894.—It is but a very short time since we received the first edition of Dr. Goodhart's book, and had the pleasure of calling attention to its excellence. We are glad to find that a second edition has been so soon required. The book is most interesting, and no one can read it without profit.

*Transactions of the Royal Academy of Medicine in Ireland.* Vol. XI. Dublin: FANNIN AND COMPANY. 1893.—We have again to notice the continued evidence of activity and progress displayed by the Irish Academicians in Medicine. This volume contains a long list of papers on a great variety of topics presented in a readable form and well worthy of study.

*Transactions of the American Surgical Association.* Vol. XI. Pp. xx., 372. Philadelphia: WILLIAM J. DORNAN. 1893.—We always take up with pleasure the annual volume of the *Transactions of the American Surgical Association*, knowing that we shall find it a storehouse of good work, and the present volume has not disappointed us. It opens with a valuable paper by Nicholas Senn on "A New Method of Direct Fixation of the Fragments in Compound and Ununited Fractures," wherein the writer, after reviewing various former methods, advocates a plan which he has made use of in three cases, of uniting the divided ends of the bone by a ferrule prepared from ox-bone. In December, 1893, we directed attention to the details of his plan. Other papers in this volume include one by William White on "The Present Position of the Surgery of the Hypertrophied Prostate;"

"A Report of Cases of Operative Attack upon Meckel's and the Gasserian Ganglions," by Roswell Park; a paper on "Gunshot Wounds of the Intestines," by Albert B. Miles; and one on "The Surgery of the Rectum," by Arpad G. Gerster. All these are marked by that originality and freedom from traditional grooves so characteristic of American surgery, and give abundant evidence of the good work being done on the other side of the Atlantic. The interest of the papers is much increased by short reports of the discussions which followed their reading.

*Transactions of the New York Academy of Medicine.* Vol. IX. Pp. xii., 408. 1893.—This volume is as full of interesting topics as previous ones have been. Of the twenty-nine papers which make up the whole, the first seven are devoted to tuberculosis. Dr. T. Mitchell Prudden discusses "The Element of Contagion in Tuberculosis"; Dr. E. G. Janeway deals with "The Necessity and Feasibility of Efforts to Prevent the Spread of Pulmonary Tuberculosis"; Dr. J. S. Billings gives statistics on the "Prevalence of Consumption in the United States"; Dr. C. G. Currier contributes a paper on "The Origin and Restriction of Tuberculosis"; Dr. J. West Roosevelt indicates the "Practicable and Impracticable Plans for Diminishing the Spread of Phthisis Pulmonalis"; Dr. V. P. Gibney gives some "Final Results in Tubercular Ostitis of the Knee in Children"; and various other members have given their views in discussions on these topics. Together these papers form a powerful manifesto in favour of the modern view with regard to the contagion of phthisis, and the prevention of its dissemination.

*Year-Book of the Scientific and Learned Societies of Great Britain and Ireland.* London: Charles Griffin and Company, Limited. 1894.—On turning over the pages of this book, one is struck by the large number of institutions which exist in Great Britain for the interchange and furtherance of scientific and technical knowledge. Wherever men meet together some "Society" is sure to spring into existence; and nearly all of these have been discovered and duly chronicled by the editors of this work. Whether such a compilation is entitled to praise or blame obviously depends on its accuracy and its completeness. Of the latter it is difficult to judge. Its accuracy we can best test perhaps by the reference to our own Bristol Medico-Chirurgical Society. We notice that the Presidents are stated to be "Dr. Markham Skerritt and Greig Smith." This should be explained by a note that the year 1893, to which the information in the book refers, includes the end of one session and the beginning of another, and therefore covers two official years. The statement, as it stands, would seem to indicate that two men occupy the presidential chair at once—a horrible idea. Dr. Armand Ruffer is styled Dr. W. Ruffer; and the papers read are wrongly stated to be published in the *Bristol Medico-Chirurgical Journal*. If this is a fair specimen of the rest of the book, we cannot praise it highly for its accuracy. It is evident, however, that considerable pains have been taken to make it a convenient handbook of reference.

*Saint Bartholomew's Hospital Reports.* Vol. XXIX. London: SMITH, ELDER & CO. 1893.—This volume contains numerous papers of great interest, such as we find as a rule in the reports of our large London hospitals. It is difficult amongst so many of value to refer to only a few as of any special interest. At the present time, when so much attention is given to ligature of the internal jugular vein in cases of pyæmia from septic conditions communicating with the lateral sinus, Mr. Walsham's two cases are of great interest. The paper by Mr. Waring on "Acute Infective Osteo-periostitis" should be read by everyone not thoroughly familiar with the disease, if only to have his attention called to the frequent errors made in its diagnosis from acute rheumatism. Mr. Butlin's account of a year's surgery at Saint Bartholomew's—his first year as full surgeon—is full of interest. He tried how well he could get on without using antiseptics for the wounds made by operation, but by failing to sterilise his sponges of cotton wool, his system proved very inadequate to deal with wound infection. Those who do not read our large London hospital reports miss many of the most instructive papers published during the year.

*On the Features which distinguish Epidemic Roseola (Roserash) from Measles and from Scarlet Fever.* By CLEMENT DUKES, M.D. Pp. 39. London: J. & A. CHURCHILL. 1894.—Dr. Clement Dukes, the able Medical Officer of Rugby School, has done valuable service to all the schools of England by his writings, all of which are well worthy of study. In common with most school doctors, Dr. Dukes has had much to do with the exanthemata, and he knows well by long experience the difficulties of such cases, and the dangers, inconveniences, worries, and loss of professional repute which follow from mistakes in their diagnosis. The pamphlet which is the subject of this notice is another praiseworthy effort to reduce the difficulty of diagnosis in one of these zymotic diseases to a minimum. Dr. Dukes says truly: "There are no cases in medicine which at times are more puzzling and require the expenditure of more thought and judgment than the zymotic diseases, roserash, measles, and scarlet fever. To mistake roserash for measles causes infinite trouble; but to confuse roserash with scarlet fever (as is constantly done), is a very serious error indeed; and yet they are as separable as typhus is from typhoid fever." Nevertheless he admits with commendable candour that in a group of cases which occurred at Rugby he one day diagnosed the cases as scarlet fever, the next day as roserash, and so on for a week, until he finally declared in favour of roserash. An experience very similar to this was published in the *Bristol Medico-Chirurgical Journal* in March, 1892. Dr. Dukes very properly denounces the irritating and misleading nomenclature of this disease, which has no less than seven different appellations. Is there no remedy for this ? Cannot the College of Physicians relieve this unhappy malady from such a burden. The author suggests that epidemic roseola as the technical name, and roserash as the popular, should be adopted, and that the others—rötheln, German measles, rubeola, etc., should be abolished. Dr. Dukes describes two forms of epidemic roseola. In one epidemic the whole of the cases will resemble measles; in another, all the cases will resemble scarlet fever. And we may add that many cases will begin as measles, and in a few days the rash will coalesce and cover the body with an eruption indistinguishable from that of scarlet fever. The measles variety occurs generally in large numbers, the scarlet fever variety in fewer. The most important part of this work is the clear and distinct way in which Dr. Dukes lays down the distinguishing characteristics between epidemic roseola or rose-rash and morbilli or measles on the one hand, and between epidemic roseola and scarlet fever on the other. In a manner which can be seen at a glance he places side by side the distinctive marks between these affections. Their premonitory symptoms, periods of incubation, clinical features, desquamation and duration of infectiveness are clearly contrasted. This pamphlet ought to be in the hands of every practitioner who has much to do with the treatment of children, and it will be especially useful to school doctors, for it is mostly in large schools that such epidemics of roseola are usually met with.

*The Johns Hopkins Hospital Reports.* Vol. IV., Nos. 1 [2-3]. Baltimore: THE JOHNS HOPKINS PRESS. 1894.—This number consists of eight papers on various points relating to typhoid fever. Six of them are written by Dr. William Osler, and form an important contribution to the modern literature of this disease. We observe with some astonishment that "no known drug shortens by a day the course of the fever; no method of specific treatment or of antisepsis of the bowel has yet passed beyond the stage of primary laudation." We hope for better results than this from the continued efforts of the Johns Hopkins School. Meanwhile it is gratifying to find that the cold-bath treatment, in spite of its harshness, has reduced the mortality from 24.2 to 7.1 per cent. A contribution by Dr. W. S. Thayer gives the results of a painstaking investigation of the blood in cases of post-typhoid anæmia.

1893. *City and County of Bristol: Annual Report of the Medical Officer of Health.* Bristol: BENNETT BROS., Ltd. 1894.—This annual summary calls for little comment. With regard to the 363 deaths from phthisis, Dr. Davies remarks that "there appears to be no valid reason for the continued existence of this fatal scourge in anything like its present proportions, if to the medical treatment of its symptoms and results were added some intelligent preventive [treatment on the part of the patient;" and he urges the need for notification, which would tend towards the adoption of the precautions needed, and would ensure the periodical and thorough disinfection of infected rooms and houses. A very excellent code of rules for preventing the spread of consumption follows. Would that it could be enforced! Were this possible we might well look for a great diminution in the death rate from phthisis.

*Intercolonial Quarterly Journal of Medicine and Surgery.* Vol. I. No. I. May, 1894. Melbourne: STILLWELL & Co.—We are pleased to welcome a new colonial exchange, with a large staff of editors in New South Wales, New Zealand, South Australia, Queensland, Tasmania, Western Australia, and Victoria. No. I contains 104 pages of matter and several excellent plates, of the usual quarterly journal variety, founded mostly on individual or on groups of cases, the most noteworthy being part of an essay on Dysphemia by Dr. Coxwell, of Melbourne. In addition to five original articles, and a number of clinical records, we find a periscope of nearly thirty pages and some small-type book reviews. We think that the editors require a little more confidence in their own efforts. Such a journal, conducted with energy and spirit, can only be welcomed with cordiality, and should have a wide circulation not only on the Australian continent but throughout the world.

